# Role of Artificial Intelligence in Reducing Error Rates in Radiology: A Scoping Review

**DOI:** 10.7759/cureus.91957

**Published:** 2025-09-10

**Authors:** Bhagya Sree Velamala, Shanti Gautam, Sulochan Lohani

**Affiliations:** 1 General Medicine, Betsi Cadwaladr University Health Board, Bodelwyddan, GBR

**Keywords:** artificial intelligence (ai), artificial intelligence in healthcare, artificial intelligence in radiology, error rates, scoping review

## Abstract

This scoping review examines how artificial intelligence (AI) can help reduce errors in radiology, an area where accuracy is critical to patient care. Radiology inherently involves complex image interpretation, and even minor mistakes can lead to delayed or wrong diagnoses, as well as inappropriate treatment. With advancements in AI, particularly in machine learning (ML), deep learning (DL), and natural language processing (NLP), there is growing interest in how these technologies can support radiologists and improve clinical outcomes. This review analyzed 12 studies that applied AI at various stages of the radiology workflow: before, during, and after image acquisition. AI has been used to assist in selecting appropriate imaging protocols, improving patient positioning, reducing motion artifacts, identifying abnormalities in scans, and supporting the generation of radiology reports. Across these applications, AI consistently demonstrated improvements in accuracy, sensitivity, and specificity, while significantly reducing reported error rates. In several cases, AI tools successfully flagged overlooked findings, acting as a safety net in high-pressure clinical environments. Despite these promising results, challenges remain. Issues such as algorithmic bias, limited data quality, and the need for robust clinical validation still require attention. Nonetheless, the evidence suggests that AI can serve as a valuable adjunct, enhancing diagnostic precision and supporting radiologists in delivering safer, more effective care. Overall, this review highlights the growing practical impact of AI in radiology and provides insights into how these technologies are already reshaping the field.

## Introduction and background

Radiology plays a central role in modern healthcare by providing crucial diagnostic insights that inform clinical decision-making and treatment planning. However, as with any human-driven process, radiology is prone to errors that may compromise patient safety, delay diagnoses, and negatively affect clinical outcomes [1]. These concerns have prompted increasing interest in leveraging artificial intelligence (AI) to enhance diagnostic accuracy, streamline workflows, and improve overall quality of care [1,2].

AI technologies offer promising solutions across the entire radiology workflow. Decision support systems can guide clinicians in making appropriate imaging requests based on patient-specific data, evidence-based guidelines, and clinical protocols, thereby optimising resource utilisation and reducing unnecessary or inappropriate imaging [1]. During image acquisition and reconstruction, AI algorithms assist with patient positioning, protocol selection, and image enhancement, improving anatomical visualisation and minimising errors [2].

Beyond imaging acquisition, AI has demonstrated significant capabilities in interpretation and reporting. AI algorithms and computer-aided diagnosis tools can detect subtle abnormalities, support clinical reasoning, and reduce interpretive variability among radiologists [3]. In addition, AI applications contribute to improved scheduling, automated communication, and reporting efficiencies, further enhancing patient safety and operational effectiveness [2,3].

Although numerous studies have investigated AI’s role in specific components of radiology, there is a lack of comprehensive reviews that evaluate its contributions across the entire workflow. This scoping review aims to bridge this gap by systematically exploring how AI can reduce errors at each stage - from the clinician’s initial imaging request to the final radiology report.

## Review

Materials and methods

Study Design

This study utilised a scoping review methodology to provide a broad and structured understanding of how AI contributes to reducing error rates in radiology. The review encompassed the entire radiology workflow, beginning with the initial imaging request by the physician and extending through to the generation of the final radiology report. The aim was to examine how AI can be integrated at each stage to minimise diagnostic errors and improve clinical outcomes.

Search Strategy

A systematic and comprehensive literature search was conducted to identify studies that investigated the role of AI in reducing diagnostic errors within radiology. The search strategy was guided by a clearly defined research question and applied inclusion and exclusion criteria (as shown in Table 1) to ensure relevance and quality. Databases searched included PubMed, MEDLINE, EMBASE, and Google Scholar, using a combination of keywords such as “artificial intelligence”, “radiology”, and “error rates”, along with Boolean operators to refine the results.

**Table 1:** Search keywords

Database	Keywords/MeSH terms
PubMed	artificial intelligence, diagnostic imaging and errors, radiology, accuracy in radiology, sensitivity and specificity, computer-assisted diagnosis
Embase	artificial intelligence, machine learning, deep learning, natural language processing, accuracy rate, error rate in radiology, radiology workflow
MEDLINE	accuracy rate, radiology workflow, radiology, artificial intelligence OR AI, radiology errors
Google Scholar	Al OR artificial intelligence, machine learning, reporting error, radiology errors

The inclusion criteria focused on studies that specifically reported outcomes related to error reduction and involved AI applications within radiological practice. To ensure a comprehensive search, additional sources such as conference proceedings, theses, and technical reports were reviewed systematically. Reference lists of all included studies were manually screened to identify any relevant articles that might have been missed during the initial database search, as outlined in Table 2. From the selected studies, key data were extracted, including study characteristics, types of AI techniques applied, specific stages of the radiology workflow targeted, and the impact on error rates. This information was synthesised to map the current landscape of AI integration in radiology and its potential in improving diagnostic precision.

**Table 2:** Inclusion and exclusion criteria

Aspects	Inclusion criteria	Exclusion criteria
Publication	After 2010	Before 2010
Intervention	Artificial intelligence	Other than artificial intelligence
Healthcare branch	Radiology	Other than radiology
Outcome	Improved diagnostic accuracy	Workload
Study design	All primary studies, irrespective of the study methodology	Argumentative essays
Population	Human (any age group)	Non-human, animal
Image modality	Any	-
Language	English	Non-English

Data Collection and Analysis

All identified articles were screened using predefined eligibility criteria to ensure the relevance and rigour of the included studies. Only studies that explored the use of AI algorithms, techniques, or technologies by radiologists to reduce error rates were selected. For each eligible study, essential details were extracted, such as the author’s name, year of publication, study design, AI methods employed, specific points of application within the radiology workflow, and the reported findings or outcomes.

A thematic analysis approach was used to interpret the extracted data. Recurrent patterns and key concepts across the studies were examined and categorised into major themes that reflected different stages of the radiology workflow. These thematic areas highlighted where AI interventions are currently being implemented - from the clinician’s initial imaging request to the final radiology report.

**Figure 1:**
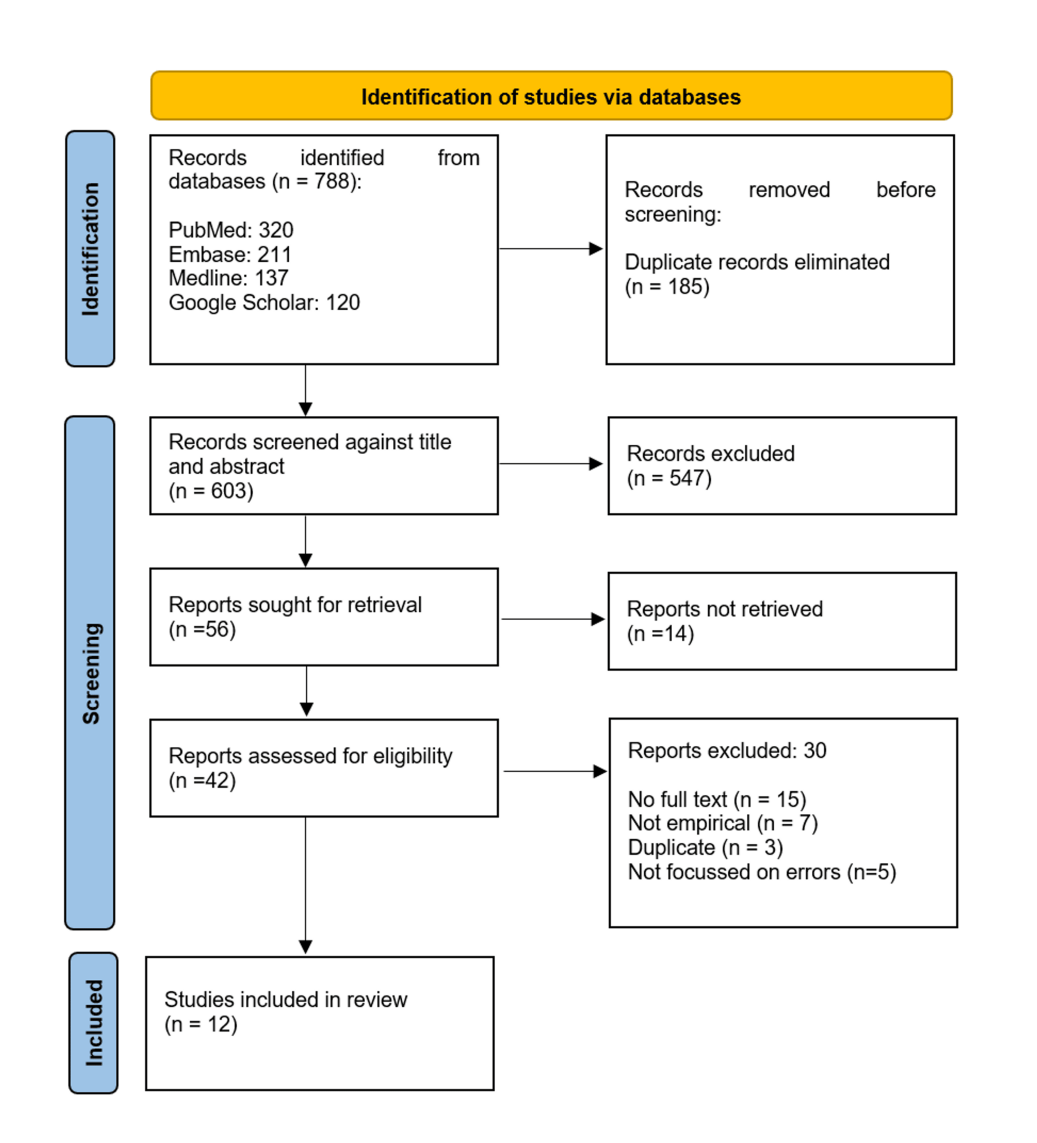
Preferred Reporting Items for Systematic Reviews and Meta-Analyses (PRISMA) flowchart for the study selection

To facilitate clarity and provide a structured understanding of AI integration, the studies were classified into three domains based on the point of AI intervention: before image acquisition, during image acquisition, and after image acquisition. The data was then organised according to these categories to illustrate the full scope of AI applications throughout the radiological process. To ensure transparency and methodological rigour, this review followed the PRISMA 2020 guidelines, as depicted in Figure 1 [4].

Ethical Considerations

This study was conducted in accordance with established ethical standards for systematic and scoping reviews. As the research involved analysis of previously published literature and did not include any human participants or primary data collection, the risk of ethical breach was minimal. All included sources were appropriately cited and acknowledged to ensure academic integrity. Ethical approval was obtained from the College of Medicine and Health’s Research Ethics Committee at the University of Exeter, under the Certificate of Ethical Clearance with reference number 148/23/07/67.

Results

Selection of Sources of Evidence

A total of 788 records were initially retrieved through searches across four databases. After removing 185 duplicate entries, 603 publications remained for the screening phase. These were assessed based on titles and abstracts, leading to the exclusion of 547 records that did not meet the inclusion criteria.

This left 56 publications for full-text assessment, of which 14 were irretrievable. Among the remaining 42 records, additional exclusions were made at the eligibility stage: 15 lacked full-text access, seven were not empirical in nature, and eight did not mention error-related outcomes. Following this multi-stage screening process, 12 studies were included in the final review for data extraction and analysis. The selection process is illustrated in the PRISMA flowchart (Figure 1) [4].

Characteristics of Sources of Evidence

The 12 included studies were categorised based on how AI was integrated within the radiology workflow. The majority of the studies focused on applications in medical imaging and radiology, with eight specifically discussing challenges and considerations for future implementation. Most of the studies were published in 2022, and retrospective study designs were the most frequently used methodology. Commonly reported aspects included the type of AI technology used, performance measurement outcomes, and validation techniques. Detailed outcome measures and summaries for each of the domains in which AI was applied are presented in Tables 3-5.

**Table 3:** Category 1: applications of AI before image acquisition

Author/year	Blackmore CC et al./ 2011 [5]	Thurston et al./2022 [6]	Muelly/2017 [7]	Brown and Marotta/2017 [8]	Lee/2018 [9]
Purpose of AI	Clinical decision-making to order imaging	Patient safety screening - pacemaker detection	Scheduling	Scan protocoling	Scan protocoling
Study design	Retrospective cohort study	Retrospective study	Observational study	Retrospective study	Observational study
AI technique	Machine learning	Machine learning	Machine learning	Machine learning	Deep learning
AI algorithms	Clinical decision systems	Neural network	Clinical decision systems	Natural language processing	Convolutional neural network
Sample size	344	300	1080	1,035	1018
Target population	Adults	Adults	Any age group	Adults	Adults
Tasks examined	CT and MRI	Chest X-rays	MRI scans	MRI brain	MRI musculoskeletal
Sensitivity	94%	100%	77.60%	83.00%	95.60%
Specificity	92.10%	99.30%	74.00%	82.90%	92.10%
Accuracy	92.67%	99.67%	75.10%	88.20%	94.20%
Reported error rate before AI application	26.00%	32.00%	54.00%	44%%	22.00%
Reported error rate after AI application	8.00%	10%%	26%	16%%	8%%
Results	The incorrect selection of advanced imaging tests has been significantly decreased because of the use of machine learning.	Automatic device screening made possible by neural networks can be scheduled before an MRI appointment to increase assurance and schedule safety checks.	The high false-positive rate emphasises the importance of improving accuracy to reduce unnecessary tests and improve appropriate tests.	NLP models can be used to predict the protocol and priority of MRI brain scans. The potential use of NLP for the MRI brain protocol increases diagnostic effectiveness, quality, and cost.	The findings back up employing deep learning to give radiologists rapid and highly accurate protocol determinations to help them in their work. Additionally, CNN-based text learning and applications could be expanded to additional radiologic tasks besides image interpretations, permitting increased work performance for radiologists, and minimising human errors in radiology, including laterality errors of radiologic reports.
Comments	Limited evidence because of the narrow demographic focus. Retrospective study design with possible biases, which are data quality issues, interpretability of the algorithms, and the difficulties with clinical integration and long-term evaluation.	The generalisability of a sample of 300 persons may be constrained. Retrospective design adds biases, and real-world applicability and clinical implementation issues were not addressed.	The study shows that machine learning can be used to schedule MRI scans, but the high false-positive rate points to the need for improvement to increase accuracy and cut down on pointless tests.	The study has some limitations, including a small sample size (1,035 people), limited generalisability, potential bias in retrospective data, and difficulties addressing natural language diversity for NLP models.	The study included a small sample size (1,018 adults), potential biases in data collection, and difficulties applying results to other populations.

**Table 4:** Category 2: applications of AI during image acquisition

Author/year	Gang et al./2021 [10]	Kromrey et al./2020 [11]	Yu et al./2022 [12]
Purpose of AI	Patient positioning	Image optimisation - motion artifact reduction	Image optimisation - segmentation
Study design	Observational study	Retrospective study	Cohort study
AI technique	Deep learning	Deep learning	Deep learning
AI algorithms	Neural network	Neural network	DR-UNET
Sample size	127	192	562
Target population	Adults	Adults	Adults
Tasks examined	CT chest	MRI abdomen	CT brain
Sensitivity	99.10%	72.10%	86%
Specificity	98.60%	70.00%	82.20%
Accuracy	99.90%	76%	88.00%
Reported error rate before AI application	26.30%	67%	35.54%
Reported error rate after AI application	6%	32%	18%
Result	The use of AI-based autonomous patient positioning and centring leads to lower radiation exposure, improved diagnostic effectiveness, higher positioning accuracy, and higher image quality.	Minimising motion artefacts using deep learning improves diagnostic accuracy in subjects who fail breath-hold in the scan.	A deep learning model called DR-UNet greatly assisted in segmenting haematomas, providing precise and reliable predictions even in irregular and unique situations, and minimising rescanning and segmentation mistakes.
Comments	The study only involved COVID-19 patients and had a small number of participants overall.	The simulation ran under the restrictive condition of respiratory motion, and the simulated datasets were not generalised to cover all scenarios.	Only 562 adults made up the sample, which was relatively small and could limit how far the results can be applied. Second, the study was restricted to CT brain scans; therefore, the findings might not generalise to other imaging modalities or anatomical areas.

**Table 5:** Category 3: applications of AI after image acquisition

Author/year	Lin et al./2019 [13]	Cheik et al./2022 [14]	Casey et al./2021 [15]	Hayes et al./2014 [16]
Purpose of AI	Image interpretation	Image interpretation	Reporting errors	Incidental findings
Study design	Retrospective study	Retrospective study	Retrospective study	Retrospective study
AI technique	Deep learning	Deep learning	Machine learning	Machine learning
AI algorithms	Neural network	Neural network	Natural language processing	PACS-based alert system
Sample size	818	1202	164	158
Target population	Adults	Adults	Any age group	Adults
Tasks examined	MRI nasopharynx	CTPA	Mixed	Chest radiography
Sensitivity	96%	94%	89%	92%
Specificity	94.23%	92.33%	86%%	89.46%
Accuracy	99.00%	98.23%	98.67%	96%
Reported error rate before AI application	22%%	25.65%%	12.00%	33%
Reported error rate after AI application	6%	8%	9%	12%
Result	The results demonstrate that the use of AI assistance can significantly increase contouring accuracy while lowering intra-and inter-observer error and contouring time. These improvements may have a positive influence on tumour control and patient survival.	AI for pulmonary embolism detection looks to be a safety net in emergency radiology practice due to excellent sensitivity and negative predictive value.	The retrospective analysis of the use of NLP to radiology reports from 2015 to 2019 indicated a significant increase in research effort, but no obvious improvement in the reporting of crucial data.	The development of electronic alert systems that are incorporated into PACS can significantly improve report communication and minimise the risk of leaving unread reports that include important or unexpected results.
Comments	Limited sample size (818 adults), only focused on MRI nasopharynx, no comparison with non-AI methods, and potential bias due to retrospective design. Further validation and broader studies are needed.	The study was based on a single aspect. No mention of whether it could be applied to other clinical conditions.	Limited sample size and lack of comparison between AI and non-AI methods.	Small sample size and limited to chest radiography

Domain of Use

To better understand the implementation of AI in radiology, the included studies were grouped according to the phase of the workflow in which AI was applied - from the physician’s initial imaging request to the generation of the final radiology report. Based on process mapping, the studies were classified into three distinct categories: (1) before image acquisition, (2) during image acquisition, and (3) after image acquisition. These domains provide a comprehensive framework to examine how AI contributes to reducing error rates across different stages of radiological practice. An overview of the 12 representative studies within these categories is provided in the corresponding tables.

Category 1: Before Image Acquisition

Five studies demonstrated how AI models can enhance various pre-imaging processes, including scan protocoling, imaging test selection, and patient safety screening. A retrospective cohort study by Blackmore et al. [5] investigated clinical decision-making in imaging selection. The incorporation of clinical decision support tools resulted in a reduced error rate of 8.0%, while simultaneously improving diagnostic accuracy and resource utilisation in a cohort of 344 adult patients undergoing CT and MRI scans.

Thurston et al. [6] focused on patient safety screening by evaluating the effectiveness of machine learning (ML) and neural networks in identifying pacemakers from chest X-rays. The model achieved excellent performance, reporting 100% sensitivity and 99.30% specificity, with an associated error rate of 10%. Similarly, an observational study by Muelly et al. [7] assessed 1,080 patients undergoing MRI scheduling using ML-based clinical decision algorithms. While the false-positive rate was relatively high at 26%, the overall accuracy reached 77.60%, emphasising the need for refinement in order to avoid unnecessary testing and to enhance diagnostic relevance.

Two additional studies addressed the use of AI in protocol selection. Brown and Marotta [8] conducted a retrospective analysis using natural language processing (NLP) to predict appropriate MRI brain scan protocols. The model achieved a prediction accuracy of 83.00%, with a reported error rate of 16%. In another observational study, Lee [9] applied DL with convolutional neural networks to optimise protocoling for MRI musculoskeletal examinations. The results demonstrated high performance, with an accuracy rate of 95.60%, specificity of 92.10%, sensitivity of 94.20%, and an overall post-AI error rate of just 8%.

Category 2: During Image Acquisition

Three studies explored the contribution of AI to improving the image acquisition process. Gang et al. [10] carried out an observational study involving 127 COVID-19 patients undergoing CT chest imaging with AI-based autonomous patient positioning. The AI-enhanced positioning approach achieved an impressive 99.90% accuracy, with an associated error rate of 6%. The study highlighted the benefits of this technology, including reduced radiation exposure, enhanced diagnostic efficiency, and improved image quality through better positioning accuracy.

A separate retrospective study by Kromrey et al. [11] examined the application of deep learning (DL) algorithms to reduce motion artefacts in abdominal MRI scans of 192 adult patients who were unable to hold their breath. The AI-assisted technique resulted in a significant 72.10% reduction in motion artefacts, thereby improving diagnostic clarity and reliability.

In a cohort study involving 562 patients undergoing CT brain scans, Yu et al. [12] employed a DL model known as DR-UNET to optimise image segmentation. The model accurately segmented haematomas with 86% precision, producing consistent results even in atypical or challenging cases. This reduced the need for rescanning and significantly decreased segmentation-related errors.

Category 3: After Image Acquisition

Post-acquisition AI applications, particularly in image interpretation and radiology reporting, were assessed across four retrospective studies. Lin et al. [13] explored AI-assisted contouring in MRI scans of the nasopharynx in a study involving 818 adult participants. The AI model achieved 96% contouring accuracy and helped minimise intra- and inter-observer variability. Additionally, contouring times were reduced, which may indirectly improve tumour control and patient survival rates.

Cheikh et al. [14] conducted a retrospective study on 1,202 adult patients focusing on the interpretation of CT pulmonary angiography (CTPA) images. The incorporation of AI tools improved both the speed and accuracy of image reading, yielding a 94% accuracy rate and reducing the error rate to 8%.

Casey et al. [15] examined the use of NLP in the generation of radiology reports. While NLP was highly accurate in extracting key clinical information, achieving an accuracy rate of 98.67%, the overall improvement in the quality of reporting was found to be limited.

Hayes et al. [16] evaluated an ML-based alert system integrated within the Picture Archiving and Communication System (PACS) for the identification of incidental findings in adult chest radiography. The model achieved a sensitivity of 92%, a specificity of 89.46%, and an overall accuracy of 96%, with a reported error rate of 12%. These findings suggest a promising role for AI in enhancing communication and follow-up of incidental but clinically important findings.

Discussion

AI holds substantial promise for improving radiology practices and patient management. However, its real-world clinical impact has thus far been demonstrated in a relatively limited number of cases. Much of the current evidence is derived from retrospective analyses or simulations rather than from large-scale prospective studies. This limitation may be partly due to the early developmental stage of the field. On average, healthcare innovations take approximately 17 years to be widely adopted into clinical practice [17], suggesting that AI’s full integration into radiology remains in its infancy.

This scoping review examined the role of AI in reducing radiologic errors. From an initial pool of 783 articles, 12 studies were ultimately included. These studies provided insights into two primary areas: the influence of AI on patient safety and clinical quality of care, and its broader implications for healthcare management.

Diagnostic errors can significantly affect clinical outcomes by leading to delayed or incorrect diagnoses, inappropriate treatments, and compromised patient safety. AI systems can serve as a secondary reviewer, increasing the accuracy and consistency of radiological interpretations and helping to reduce diagnostic errors [2]. For instance, clinical decision support tools have been shown to improve physician decision-making and patient scheduling by minimising errors in imaging modality selection. This led to shorter patient wait times, optimised scheduling, reduced examination costs, and improved patient satisfaction [5].

In another example, neural networks identified pacemakers on radiographs with an accuracy of 99.67% [6]. AI has also enhanced operational efficiency in scheduling - particularly for high-demand imaging modalities such as CT and MRI - by mitigating human limitations in interpreting incomplete or unclear clinical information. AI-driven protocols improved scheduling quality, consistency, and efficiency by tailoring imaging workflows to clinical needs [8]. These optimisations not only streamlined imaging operations but also showed potential to enhance patient safety and clinical outcomes.

In terms of image quality, AI has facilitated advancements that were previously limited by manual processes. For example, lesion contouring, which is traditionally too time-intensive for routine clinical use, can be automated through AI technologies, enabling consistent quantification and segmentation. This approach holds the potential to integrate advanced image analytics into daily radiological practice [12]. Nevertheless, further research is necessary to validate the consistency, prognostic accuracy, and broader clinical utility of these AI-driven techniques.

Report generation in radiology may also benefit from AI applications, particularly through the use of NLP. Beyond improving speech recognition, these systems can review prior reports to prompt radiologists about common errors, such as referencing the incorrect side or omitting a previously noted finding, such as an incidental adrenal nodule. A study demonstrated the value of a PACS-integrated AI tool capable of flagging incidental findings and assisting in retrieving follow-up data. Such tools allow radiologists to reflect on prior interpretations, thus supporting continuous improvement in reporting accuracy [16].

In addition to direct clinical benefits, the integration of AI into radiology introduces important considerations for healthcare administration. Implementing AI solutions requires substantial investment in infrastructure, particularly in high-performance computing systems and robust data storage solutions. Healthcare organisations must plan strategically and allocate sufficient resources to support not only the initial implementation but also the ongoing maintenance and optimisation of AI systems. Effective adoption also demands that radiologists and clinical staff receive targeted training and education. Institutions must prioritise structured training programmes and workforce development to ensure that AI tools are used competently and ethically within clinical workflows [18].

Infrastructure readiness is critical, as AI algorithms must process large volumes of medical imaging data in real time. Healthcare providers need to assess current technological capabilities and make informed decisions regarding upgrades and expansions. Moreover, given the rapid pace of advancement in AI technologies, it is essential that organisations remain agile and informed. This may involve forming partnerships with technology vendors, collaborating with academic institutions, or establishing in-house AI development teams.

The long-term success of AI integration depends on its sustainability and scalability, which should be embedded into financial and operational planning. Ongoing professional development is essential to ensure that radiologists and healthcare providers remain equipped to adapt to evolving AI capabilities and continue to deliver safe, high-quality patient care [18].

Limitations

While this scoping review employed rigorous methods, several limitations must be acknowledged. Firstly, the quality of included studies was not formally assessed, which may influence the strength and reliability of the review’s conclusions. Although a broad search strategy was used across multiple databases, it is possible that some relevant studies were overlooked. In addition, limiting the search to English-language articles published after 2010 may have excluded earlier or non-English studies of significance. These constraints could affect the comprehensiveness of the findings. Nevertheless, the review offers valuable insights into the current landscape of AI applications in radiology and identifies key areas for future research.

## Conclusions

This scoping review provides a comprehensive analysis of the role AI plays in reducing error rates in radiology. By systematically reviewing current literature, the study highlights how AI is being integrated at various stages of the radiology workflow - from the physician's imaging request to the final report. The findings suggest that AI technologies have the potential to significantly enhance diagnostic accuracy, improve patient outcomes, and streamline administrative functions. Advanced algorithms and image analysis techniques enable AI systems to detect subtle abnormalities and support radiologists in delivering faster, more accurate diagnoses.

Despite these promising developments, the implementation of AI in clinical practice is not without challenges. Key concerns such as data quality, patient privacy, and regulatory compliance must be addressed to ensure safe and ethical use. As AI technologies continue to evolve, their collaboration with radiologists is expected to become more seamless, driving a transformative shift in the field. However, it is crucial to maintain a balanced approach - recognising AI as a complementary tool that augments human expertise rather than replaces it.
